# KIM-1/TIM-1 in proximal tubular cell immune response

**DOI:** 10.18632/oncotarget.6623

**Published:** 2015-12-15

**Authors:** Craig R. Brooks, Joseph V. Bonventre

**Affiliations:** Renal Division, Department of Medicine, Brigham and Women's Hospital, Harvard Medical School, Boston, Massachusetts, USA and Harvard Stem Cell Institute, Cambridge, Massachusetts, USA

**Keywords:** KIM-1, TIM-1, phagocytosis, MHC presentation, NFκB

**Figure 1:**
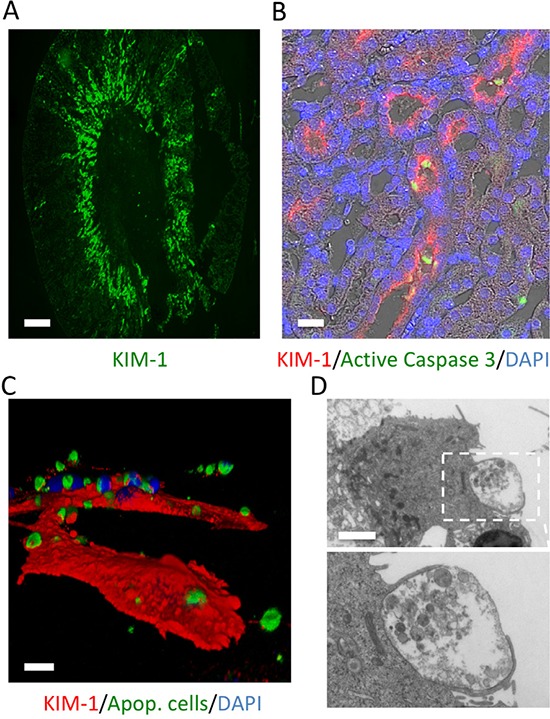
KIM-1 is expressed by PTCs and mediates apoptotic cell phagocytosis Following bilateral kidney ischemia in mice, KIM-1 is primarily expressed in the PT S3 segment, the area of most severe injury (A), where it localizes to the brush border and binds caspase 3 positive cells (B). *In vitro*, 3D reconstruction (C) or electron microscopy (D) reveals KIM-1 expressing LLC-PK1 cells actively phagocytose apoptotic cells. (Scale bars: A, 500 μm; B, 50 μm; C, 10 μm; D, 2 μm) (D, published previously [6]).

Kidney injury molecule-1/T cell IgG and mucin containing 1 (KIM-1/TIM-1) is a type 1 membrane receptor [1] which we identified as the most upregulated proximal tubular cell (PTC) protein following a variety of kidney injuries in animal models and human diseases [1] (Figure 1A). While KIM-1 (also known as hepatitis A virus cellular receptor-1 (HAVCR-1)) quickly was recognized as a promising biomarker, other proteins were subsequently discovered which, together with KIM-1/TIM-1 formed a new class of proteins (TIMs) whose function was unknown at the time of discovery. Work from our lab identified KIM-1′s function as an apoptotic cell phosphatidylserine phagocytosis and scavenger receptor [2], whereby it induces the binding and uptake of dead cells from the kidney tubule lumen in acute kidney injury (AKI) (Figure 1B). As is often the case, however, identifying the function of KIM-1 only led to more questions: Why do non-myeloid cells express a scavenger receptor? Can PTCs efficiently phagocytose *in vivo*? What are the functional consequences of PTC phagocytosis? In other words, what is the role of KIM-1 in kidney injury?

To address these questions, we characterized a mouse model KIM-1^Δmucin^ (in collaboration with Dr. Vijay Kuchroo's lab), which expressed a truncated, phagocytosis deficient KIM-1 molecule, by deleting the mucin domain, (the domain where most mutations leading to human disease occur), while retaining most of the protein intact [3]. Utilizing this novel animal model, we found that KIM-1 mediated phagocytosis is responsible for the clearance of much of the luminal apoptotic cells resulting from ischemic or nephrotoxic acute tubular injury [4]. In addition, KIM-1 expression down-regulates PTC cytokine secretion, which is further down-regulated with the KIM-1-mediated uptake of apoptotic cells. Using a systems biology approach, we identified post-translational modifications to the NFκB pathway as the most likely pathway modulated by KIM-1 to regulate cytokine secretion [4]. Indeed, KIM-1 expression and phagocytosis decreases NFκB phosphorylation and activity. KIM-1 phosphorylation regulates the NFκB pathway through interaction and regulation of the PI3 kinase subunit p85. In the KIM-1^Δmucin^ mice, decreased apoptotic cell clearance, increased pro-inflammatory cytokine secretion and increased immune cell infiltration culminated in more severe injury, when compared to wild-type mice, indicating intact KIM-1 plays a protective role in AKI [4].

In addition to acting as non-professional phagocytes, PTCs constitutively express MHC II and can activate naïve T cells; thus, PTCs are also semi-professional antigen presenting cells [5]. To determine if KIM-1-mediated phagocytosis promotes presentation of phagocytosed antigens, we first examined the uptake and processing of apoptotic cells *in vitro* by adding apoptotic cells to the apical surface of cultured PTCs and monitoring phagocytosis (Figure 1C). Apoptotic cells taken up by KIM-1 are quickly targeted by LC3 in the cytosol. LC3 localization to KIM-1 phagosomes required the expression of autophagy factors ATG5, Beclin1 and ULK1 [6]. Mutation to the KIM-1 ligand-binding domain (WFND, amino acids 115–118 in the mouse), deletion of the mucin domain or deletion of the cytosolic domain (containing the phosphorylation sites) prevents autophagy induction [6]. KIM-1 phosphorylation and interaction with p85 are critical to its regulation of autophagy, with KIM-1-induced autophagy being suppressed in p85 knockout cells [6]. Unlike professional phagocytes, KIM-1-mediated phagocytosis in PTCs resulted in slower acidification of the phagosome (compared to macrophages and dendritic cells) and did not induce dependent reactive oxygen species production [6]. Inhibition of NOX by diphenylene iodonium did not prevent LC3 localization to the phagosome [6]. These biochemical data support the role of autophagy in the clearance of KIM-1 phagosomes.

We next examined if phagocytosed material was presented to MHC I and II. KIM-1 expressing PTCs present antigens at a higher level than did KIM-1^Δmucin^ PTCs [6]. Despite the increase in antigen presentation, KIM-1 expressing PTCs are associated with inhibition of CD4+ T cell proliferation and activation and an increase in the number of T regulatory cells (Tregs) [6]. Removal of the Treg population reverses the ability KIM-1-dependent antigen presentation to inhibit T cell activation. Deletion of autophagy factors ATG5 and ULK1 also results in a pro-inflammatory MHC presentation [6]. *In vivo*, kidneys from wild-type mice have greater numbers of Tregs and reduced numbers of CD4+ and CD8+ T cells, compared to KIM-1^Δmucin^ mice [6]. Thus, material phagocytosed by KIM-1 and processed via autophagy are presented to MHC I and II, creating a pro-tolerogenic milieu.

Our earlier work demonstrated that long-term expression of KIM-1, even at low levels, is maladaptive, inducing spontaneous and progressive interstitial inflammation, fibrosis and chronic kidney disease in the mouse [7]. In models of severe AKI which progress to CKD, KIM-1 is expressed at high levels during the acute phase and remains elevated through the transition to CKD [7]. Therefore, the induction and progression of fibrosis by KIM-1 could help explain the AKI to CKD transition seen in human kidney diseases and animal models.

What can explain the dichotomy between the protective role of KIM-1 in acute and maladaptive response in chronic kidney injuries? We believe the key to answering this question lies in the differences in the pathophysiology of acute *vs* chronic injury. AKI is associated with an abundance of apoptotic cells and necrotic debris, while CKD is associated with a more mild but sustained injury, breakdown of the glomerular filtration barrier, and marked increases in modified serum proteins, lipids and other factors entering into the tubule lumen which can be taken up by KIM-1 expressing cells [7]. These ligands for KIM-1 likely exert increasing levels of stress to the epithelial cell with a resultant generation of pro-fibrotic cytokines, culminating in inflammation, activation of interstitial cells, increased matrix production, vascular rarefaction and chronic kidney disease (CKD). In contrast to the uptake of apoptotic cells leading to KIM-1 phosphorylation and autophagy, we suggest that KIM-1 ligands found in glomerular filtrate in CKD do not induce autophagy. This is perhaps due to the fact that uptake of these ligands activate a distinctive KIM-1 signaling program which promotes inflammatory processes including cytokine production and pro-inflammatory antigen presentation.

Taken together, these studies demonstrate that KIM-1 acts as a “double-edged sword” in the injured kidney. In acutely injured kidneys, KIM-1 facilitates repair by removal of luminal debris, leading to anti-inflammatory antigen presentation and down-regulation of the PTC immune response via NFκB suppression. By contast, in the chronically injured kidney, prolonged KIM-1 expression stimulates inflammation and fibrosis. Future studies are required to elucidate whether KIM-1′s phagocytic function to remove lumenal cellular debris or its ability to down-modulate inflammation is most important for its protective effect in AKI. The development of phospho-specific antibodies would enable the further exploration of divergent KIM-1 signaling in AKI and CKD, shedding light on potential mechanisms of KIM-1 action in the chronic setting. It is evident, however, that small molecules or biologics which can up or down-modulate KIM-1 activity should be explored as potential therapeutics in AKI and CKD.
